# Intensivists' base specialty of training is associated with variations in mortality and practice patterns

**DOI:** 10.1186/cc8227

**Published:** 2009-12-29

**Authors:** Emma O Billington, David A Zygun, H Tom Stelfox, Adam D Peets

**Affiliations:** 1 Department of Medicine, Foothills Medical Centre - North Tower, 9th floor, 1403 - 29th St NW, Calgary AB, T2N 2T9, Canada; 2 Department of Critical Care Medicine, Rm EG 23, 1403 - 29th ST NW, Calgary AB, T2N 2T9, Canada; 3 Division of Critical Care Medicine, Rm 239 Comox Building, St Paul's Hospital, 1081 Burrard St, Vancouver BC, V6Z 1Y6, Canada

## Abstract

**Introduction:**

Current evidence regarding whether the staffing of intensive care units (ICUs) with a trained Intensivist benefits patient outcomes is discordant. We sought to determine whether, among certified Intensivists, base specialty of training could contribute to variation in practice patterns and patient outcomes in ICUs.

**Methods:**

The records of all patients who were admitted to one of three closed multi-system ICUs within tertiary care centers in the Calgary Health Region, Alberta, Canada, during a five year period were retrospectively reviewed. Outcomes for patients admitted by Intensivists with base training in General Internal Medicine, Pulmonary Medicine, or other eligible base specialties (Anesthesia, General Surgery, and Emergency Medicine combined) were compared.

**Results:**

ICU mortality in the entire cohort (n = 9,808) was 17.2% and in-hospital mortality was 32.0%. After controlling for potential confounders, ICU mortality (odds ratio (OR): 0.69; 95% confidence interval (CI): 0.52 to 0.94) was significantly lower for patients admitted by Intensivists with Pulmonary Medicine as a base specialty of training, but not ICU length of stay (LOS) (coefficient: 0.11; -0.20 to 0.42) or hospital mortality (OR: 0.88; 0.68 to 1.13). There was no difference in ICU or hospital mortality or length of stay between the three base specialty groups for patients who were admitted and managed by a single Intensivist for their entire ICU admission (n = 4,612). However, we identified significant variation in practice patterns between the three specialty groups for the number of invasive procedures performed and decisions to limit life-sustaining therapies.

**Conclusions:**

Intensivists' base specialty of training is associated with practice pattern variations. This may contribute to differences in processes and outcomes of patient care.

## Introduction

Over the past decade, the literature has suggested that Intensive Care Units (ICUs) staffed by physicians certified in critical care medicine led to improved patient outcomes [1]. However, a recent retrospective review of over 100,000 ICU admissions found the opposite: patients managed by critical care physicians were at increased risk of death compared to those managed by physicians without critical care training [2]. Potential explanations given for these discrepant results included inability to control for unmeasured confounders and variation in physicians' practice patterns such as compliance with evidence-based protocols and use of invasive procedures.

Practice pattern variation has been attributed to many factors, including patient case mix and severity of illness, availability of resources and characteristics of the individual physician themselves [3,4]. One physician characteristic, base specialty of training, has been evaluated in non-ICU settings and found to be associated with differences in resource utilization and patient outcomes [4-6]. Within the specialty of critical care medicine there is considerable variability in base specialty of training for Intensivists, from internal medicine with or without additional pulmonary training, to anesthesia, to the surgical specialties. The training programs that serve as points of entry into a critical care fellowship vary considerably in terms of scope and focus, which may result in considerable diversity in practice styles within the population of practicing Intensivists. However, to our knowledge, the effect that this core training has on patient management and outcomes in the ICU has not previously been investigated.

Therefore, we sought to determine the effect of Intensivists' base specialty of training on practice patterns and patient outcomes in the ICU.

## Materials and methods

### Study Design

The Calgary Health Region (CHR) (population 1,197,848 as of 2006) contains three closed medical-surgical ICUs, each in academic centers affiliated with the University of Calgary. While all units manage critically ill medical and surgical patients, certain services have been regionalized. One unit is a trauma/neurosurgical referral centre with 25 beds, the second a vascular surgery referral centre that has 14 beds, and finally a 10-bed medical-surgical unit. Each ICU is staffed by attending physicians who are board-certified in critical care medicine, and do one week shifts at a time. Registered nurses are typically assigned one patient each, but may look after two patients if short-staffed.

When on service, Intensivists perform daily bedside rounds. While residents and fellows have input on the decision-making process, attending Intensivists have full responsibility for development and implementation of the daily healthcare plan on each patient in the ICU. Intensivists are on-call 24 hours per day, with call being performed from home at night. They regularly return during the night to oversee trainees. Residents from nearly every training program in the CHR, ranging from Postgraduate Year (PGY) 1 to PGY 4, complete rotations in each ICU and perform in-house overnight call. Every night has resident coverage, with residents averaging call once every fourth night. Approximately 50% of the year, an ICU fellow will also be on service at each of the sites, and will complete call from home once every three nights. Decisions to perform invasive procedures are made in conjunction with the Intensivist and depending on the experience level of the trainee, the Intensivist may or may not directly supervise the procedure. A record of all procedures is documented in the ICU electronic database, TRACER.

All patients admitted to CHR ICUs between August 1, 2002 and July 31, 2007 were identified from TRACER. If a patient was admitted to ICU more than once during the study period, one of the visits was randomly selected to be included in the analysis. During the study period, there were no major changes to the Regional Healthcare System that affected how care was delivered in the ICU.

ICU physicians were classified by their base specialty of training into one of three groups: Internal Medicine (Internal Medicine Group), Internal Medicine plus a fellowship in Pulmonary Medicine (Pulmonary Group), or Anesthesia, General Surgery and Emergency Medicine, which due to small numbers were analyzed together (AGSEM group). Over the study period three Intensivists left Calgary and six were hired.

Patients were grouped according to the base specialty of the Intensivist who admitted them to the ICU, and outcomes were compared between these groups. The primary outcome measures were ICU mortality and length of stay (LOS). We elected to use these as primary outcomes instead of the more traditional hospital mortality and LOS in order to focus on the outcomes that would maximally reflect the care provided by Intensivists and attempt to minimize effects of other variables that may influence outcomes outside of the ICU. Secondary outcomes consisted of in-hospital mortality, hospital LOS, number of invasive procedures performed and limitation of life support therapies, as judged by the number of patients changed from full care to do not resuscitate (DNR) during their ICU admission. The following invasive procedures were tracked: endotracheal intubation, chest tube, thoracentesis, central line, arterial line, pulmonary artery catheter insertion, lumbar puncture, bone marrow biopsy and paracentesis. Most procedures are done by housestaff, but direct or indirect supervision is provided by the attending Intensivist in the majority of cases.

In analysis of the entire cohort, only the identities of the admitting physicians were accounted for, despite the fact that many patients were cared for by more than one Intensivist while in ICU. *A priori*, we made the decision to also complete a subgroup analysis on those patients who were admitted and managed by a single Intensivist for their entire ICU admission in order to provide a more specific analysis of the impact that each Intensivist group may have on patient outcomes.

### Analysis

Means for continuous data were compared using the Kruskal-Wallis test or one-way analysis of variance (ANOVA) where appropriate. Categorical data was compared with use of Fisher's Exact test.

Given the significant heterogeneity in baseline patient and Intensivist characteristics, the use of regression analysis was appropriate. However, typical regression models are unable to account for clustering of patients, so we utilized generalized estimating equations (GEE) to control for correlation between individual observations. For these analyses, two sources of correlation were identified and accounted for in each model: those related to the hospital site the patient was admitted to and those related to the individual physician who cared for the patient. To evaluate variables associated with ICU and hospital mortality, we used a model built on a binomial distribution with a logit link function. As ICU and Hospital LOS were skewed, they were natural-log transformed to approximate statistical normality, and subsequently entered into separate linear scale response models with identity as the link function. Evaluation of number of procedures performed utilized GEE based on a Poisson distribution, while the model for change in level of care was built on a binomial distribution.

Given the size of the cohort, all relevant variables felt to potentially impact the dependent variable in each of the models were included [7]. Therefore, the following independent variables were included in all of the models: patient age, gender, Acute Physiology and Chronic Health Evaluation II (APACHE II) score, mean Therapeutic Intervention Scoring System (TISS) over first 24 hours of admission to ICU, year of admission, time of year of admission (by 28-day block to coincide with trainees' length of rotation), level of care at time of admission (full care or DNR) and discharge, admission diagnosis, Intensivist gender, Intensivist base specialty of training, years since completion of Critical Care Medicine Fellowship, and ICU occupancy at admission and at discharge. In addition, the number of invasive procedures performed per patient was included as an independent variable in all models except the one where it was the dependent variable, and ICU LOS was included as an independent variable in models assessing ICU and hospital mortality, number of invasive procedures performed, and the change in level of care. Separate analyses with adjustment for the variables listed above were completed for the entire cohort and the subgroup of patients who were admitted and managed by a single Intensivist. Detailed results of these analyses are provided in Additional file 1.

All *P *values < 0.05 were considered significant. Statistical analysis was done using Stata version 8.0 (College Station, Texas, USA) and SUDAAN version 9.0 (RTI International, Raleigh, North Carolina, USA). Prior to initiation of this study, ethical approval was obtained from the Conjoint Health Research Ethics Board at the University of Calgary. Permission for waiver of consent was obtained as this was a retrospective review of a database and all data was made anonymous at the time of acquisition from TRACER.

## Results

During the study period 9,808 patients had 11,663 ICU admissions, with 1,283 patients being admitted more than once. The mean patient age of the final cohort was 56.8 years; most were male (57.8%) with a mean admission APACHE II score of 23.2 (Table 1). A total of 26 Intensivists (92% male) admitted patients during the study period. Their base specialties of training were Internal Medicine (n = 12), Pulmonary (n = 8), and AGSEM (n = 6) (Table 2). Each had completed further training in Critical Care Medicine, with 23 completing dedicated multidisciplinary critical care fellowships and three surgical critical care fellowships. There were significant differences in both the baseline characteristics of the study cohort and the subgroup of patients cared for by a single Intensivist for their entire ICU stay (n = 4,612) according to physician base specialty (Table 1).

**Table 1:** Patient characteristics

	Entire cohort of patients					Patients cared for by a single Intensivist during ICU stay				
	Overall (n = 9808)	Internal Medicine (n = 4146)	Pulmonary (n = 3906)	AGSEM (n = 1756)	*p *value*	Overall (n = 4612)	Internal Medicine (n = 1944)	Pulmonary (n = 1844)	AGSEM (n = 824)	*p *value*
Mean age in years (± SD)	56.8 (18.8)	56.0 (18.9)	58.4 (18.2)	55.2 (19.7)	<0.001	57.1 (19.2)	56.6 (19.4)	58.2 (18.6)	55.7 (19.9)	<0.01
Male (%)	57.8	58.0	56.8	59.9	NS	56.0	55.5	56.0	57.0	NS
Mean admission APACHE II (± SD)	23.2 (9.1)	23.1 (8.9)	23.4 (9.3)	22.7 (8.9)	< 0.05	22.2 (9.4)	22.5 (9.3)	21.9 (9.4)	21.8 (9.3)	NS
Mean admission TISS (± SD)	35.7 (13.3)	36.3 (13.4)	34.5 (13.1)	36.7 (13.3)	<0.001	33.1 (13.3)	33.7 (13.5)	31.7 (12.8)	34.4 (13.5)	<0.001
Admitting diagnosis (% of total admissions within each group)										
Pulmonary	30.7	30.3	32.4	28.3	<0.01	28.5	28.6	29.4	26.8	<0.01
Cardiovascular	23.4	21.0	27.3	20.3	<0.001	24.3	21.9	28.0	22.1	<0.001
Neurologic	13.6	16.7	10.2	13.9	<0.001	13.6	16.3	10.2	15.0	<0.01
Gastrointestinal	10.7	10.2	11.0	10.7	NS	11.3	11.5	11.4	10.6	NS
Trauma	9.2	10.2	5.4	15.2	<0.001	6.5	7.4	3.9	10.3	<0.001
Poisoning	5.6	4.6	6.5	6.1	<0.01	7.7	6.6	8.5	8.7	<0.01
Other	6.8	6.9	7.2	5.5	<0.01	8.0	7.7	8.6	6.6	NS
Admit level of care (% DNR)	9.5	9.4	9.8	9.1	NS	11.8	12.4	11.2	11.9	NS
Discharge level of care (% DNR)	22.3	22.7	22.4	21.2	NS	21.4	23.6	19.4	20.5	<0.01
ICU occupancy at admission (%)	84.0	83.3	84.3	85.1	<0.001	83.6	83.1	83.5	84.8	<0.05

AGSEM = Intensivists with base specialty training in Anesthesia, General Surgery or Emergency Medicine; APACHE = Acute Physiology and Chronic Health Evaluation Score; DNR = Do Not Resuscitate; ICU = Intensive Care Unit; n/a = not applicable; SD = Standard Deviation; TISS = Therapeutic Intervention Scoring System

* Reflects comparisons between the three specialty groups

**Table 2:** Physician characteristics by base specialty of training

	Overall	Internal Medicine	Pulmonary	AGSEM	*p *value*
Number of physicians	26	12	8	6	n/a
Median years since critical care medicine certification (IQR)	12 (5 to 16)	9 (4 to15)	15 (12 to 18)	7 (3 to 10)	<0.001
Mean weeks of service per year (± SD)	14.5 (5.8)	15.6 (6.0)	15.4 (5.5)	9.8 (2.9)	<0.001

AGSEM = Intensivists with base specialty training in Anesthesia, General Surgery or Emergency Medicine; IQR = Interquartile range; n/a = not applicable; SD = Standard Deviation;

* Reflects comparisons between the three specialty groups

### Entire Cohort Analysis

For the entire cohort, ICU mortality was 17.2%, in-hospital mortality was 32.0%, median ICU LOS was 2.9 days, and median hospital LOS was 13.5 days (Table 3). After controlling for baseline patient, physician and ICU characteristics, patients admitted by a physician from the Pulmonary group had significantly less chance of dying in the ICU (OR: 0.69; 95% CI: 0.52 to 0.94) compared to those admitted by the AGSEM group. There were no differences in patients' ICU LOS, or hospital mortality or LOS. The Pulmonary group performed fewer invasive procedures (OR 0.96 (0.92 to 1.0)), while Intensivists in the Internal Medicine group were more likely to change patients to DNR status (OR 1.13 (1.02 to 1.24)).

**Table 3:** Outcome measures based on physician specialty

	Entire cohort of patients					Patients cared for by a single Intensivist during ICU stay				
Measures	Overall	IM	Pulm	AGSEM	*p *value*	Overall	IM	Pulm	AGSEM	*p *value*
ICU Mortality (%)	17.2	17.9	16.0	18.0	<0.05	19.9	21.8	17.4	20.8	NS
Median ICU LOS in Days (IQR)	2.9 (1.4 to 6.7)	2.9 (1.5 to 6.8)	2.8 (1.4 to 6.6)	2.8 (1.3 to 6.6)	NS	1.7 (0.9 to 2.8)	1.7 (0.9 to 2.8)	1.7 (0.9 to 2.7)	1.6 (0.8 to 2.7)	NS
Hospital Mortality (%)	32.0	32.7	31.4	31.7	NS	33.0	36.5	28.8	34.0	NS
Median Hospital LOS in Days (IQR)	13.5 (5.7 to 30.7)	14.3 (5.7 to 32.4)	13.8 (6.1 to 30.9)	13.7 (5.9 to 32.8)	NS	8.9 (3.3 to 19.0)	8.4 (2.8 to 19.2)	8.8 (3.8 to 18.9)	8.4 (3.3 to 18.7)	NS
Patients changed from Full Care to DNR (%)	12.8	13.3	12.6	12.1	<0.05	9.6	11.2	8.2	8.6	<0.05
Median Number of Procedures (IQR)	3 (2 to 5)	3 (2 to 5)	3 (2 to 4)	3 (2 to 5)	<0.05	2 (2 to 3)	3 (2 to 4)	2 (1 to 3)	3 (2 to 4)	<0.05

AGSEM = Intensivists with base specialty training in Anesthesia, General Surgery or Emergency Medicine; DNR = Do Not Resuscitate; ICU = Intensive Care Unit; IM = Intensivists with base specialty training in Internal Medicine; IQR = Interquartile Range; LOS = Length of Stay; NS = non-significant; Pulm = Intensivists with base specialty training in Pulmonary Medicine.

* Reflects comparisons between the three specialty groups using Generalized Estimating Equations

### Subgroup Analysis

For the subgroup of patients cared for by one Intensivist during their entire ICU stay, ICU mortality was 19.9%, in-hospital mortality was 33.0%, median ICU LOS was 1.7 days, and median hospital LOS was 8.9 days (Table 3). Analyses demonstrated no differences in either ICU or hospital mortality or LOS between the three groups of specialists. However, in keeping with the entire cohort, the Pulmonary group performed fewer procedures (OR 0.94 (0.90 to 0.99)) and the Internal Medicine group transitioned more patients to DNR status (OR 1.38 (1.09 to 1.66)).

## Discussion

Since previous studies have found that board-certified Intensivists may have either a positive or negative impact on patient outcome in ICUs [1,2], we sought to examine characteristics of the physicians within this highly trained group to further explore what factors may contribute to these discrepant outcomes. The results of our study suggest that the use of invasive procedures, limitation of life support measures and ICU mortality appear to vary according to Intensivists' base specialty of training.

While our results should only be viewed as hypothesis-generating given the retrospective design of the study, there are a number of factors that make the results plausible. The first is that this is not a new phenomenon. Previous reports have suggested that physicians with training in a specific area of medicine tend to have more favorable outcomes with conditions that fall into their area of expertise than do generalists [8-12]. Since over 30% of the admitting diagnoses in our ICUs are related to the pulmonary system, the Pulmonary Medicine group may have an intrinsic advantage over the Internal Medicine and AGSEM groups. In addition, extra years spent as a trainee may provide Intensivists with Pulmonary Medicine backgrounds valuable clinical experience that helps them diagnose and manage complex ICU patients more effectively than those with Internal Medicine backgrounds.

The second is that we observed a significant difference in the propensity to limit life-sustaining therapy between the three groups. While many factors play into a decision to limit life support, it has previously been shown that the identity of the individual physician is one of the most, if not the most, important determinants [13]. Different practice patterns for limitation of life support based on Intensivist's base specialty of training have not previously been described, but should now be further evaluated.

A third factor that helps explain our results is that because the Pulmonary group performed significantly less invasive procedures than the other two groups, their patients may have been at less risk to develop potential life-threatening complications [14-16]. While we had initially hypothesized that the decrease in the number of procedures was due to members of the Pulmonary group having more years of clinical experience and their greater comfort level in diagnosing and managing patients based on clinical examination and non-invasive tests alone, this turned out not to be the case according to our statistical models because we adjusted for number of years in practice. However, the lower number of procedures performed may still be a surrogate for an overall more conservative practice pattern that may benefit their patients, but that is not easily measured by a single variable such as *years in practice*. Future research should explore other areas of potential practice pattern variation based on Intensivist base specialty of training beyond the two variables that we elected to measure in this study.

While it is plausible for a physician's training to impact their patients' outcomes, it is important to note that there are limitations with our study that need to be taken into consideration when interpreting the results. First, attributing causation is challenging because of the multiple variables that impact patient outcomes in ICUs beyond the Intensivist. Second, for patients in the entire cohort that had more than one Intensivist involved in their care, we attributed patient outcomes only to the admitting ICU attending physician. While we justified this decision based upon the evidence that the first 24 to 48 hours of a patient's care often sets the trajectory for both short and long-term patient outcomes [17-21], this did not allow us to account for management decisions made later in the ICU course by other Intensivists. We specifically performed the subgroup analysis to attempt to address this issue; however, since these patients represent less than half of the patients admitted to our ICUs, it is difficult to generalize the results.

A third limitation was our inability to control for the contribution of residents, ICU fellows and members of the multidisciplinary team to patient care; however, it has previously been shown that outcomes do not change depending on whether a critical care fellow is involved in patient care [22]. Additionally, the generalizability of our results may be limited because it was performed in three Canadian teaching hospitals and because the mix of base specialties was heavily weighted towards Internal Medicine and Pulmonary Medicine. Finally, given the size of our cohort, while some of our results are considered statistically significant, one could argue whether the differences should be considered clinically significant; the difference in the number of invasive procedures performed would be an example of this.

Taken in the context of these limitations, we believe that the results of our study still have potential implications for the way in which critical care medicine is administered. First, training programs need to be aware that a trainee's base specialty may substantially influence the knowledge and skills with which they enter a fellowship program and hence their training needs and fellowship experience. Second, given predictions of a significant shortage of Intensivists in years to come [23,24], there may be pressure to preferentially recruit trainees from base specialties with a shorter duration of training, for example Internal Medicine. While this could deliver more Intensivists to the workforce more quickly, prospective multicentre investigations are first warranted to further assess the potential impact of physicians' base specialty of training on patient outcomes. Third, although care in most ICUs is provided by a highly trained multidisciplinary team with the assistance of evidence-based protocols, individual physician leadership clearly influences patient care.

Given the potential impact that Intensivist base specialty training may have on physician practice patterns, resource utilization, patient outcomes, and future training requirements for Intensivists, further investigations are warranted to explore our findings.

## Conclusions

Our results suggest that an Intensivist's base specialty of training may impact patient outcomes and practice patterns. This may help explain the inconsistent results seen with previous investigations assessing the impact of Intensivists' care on ICU patients.

## Key messages

• Intensivists' base specialty of training is associated with practice pattern variation.

• This may be one factor that contributes to the differences in processes and outcomes of patient care.

• Further prospective investigations are warranted to explore the impact that this may have on patient care and training requirements for Intensivists.

## Abbreviations

AGSEM: anesthesia, general surgery and emergency medicine; ANOVA: analysis of variance; APACHE II: Acute Physiology and Chronic Health Evaluation II score; CHR: Calgary Health Region; CI: confidence interval; DNR: do not resuscitate; GEE: generalized estimating equation; ICU: intensive care unit; LOS: length of stay; OR: odds ratio; PGY: Postgraduate Year of training; TISS: Therapeutic Intervention Scoring System.

## Competing interests

The authors declare that they have no competing interests.

## Authors' contributions

All authors were involved in development of the research question and study design. EOB contributed to data acquisition, data analysis, and drafting of the manuscript. DAZ and HTS contributed to data analysis. ADP contributed to data acquisition, data analysis, and drafting of the manuscript. All authors revised the manuscript critically for important intellectual content and have approved the final copy.

## Supplementary Material

Additional file 1 Additional file 1 is available with the online version of this paper; it contains a total of 12 tables providing more detailed results of the analyses. Tables S1 and S2 list the variables associated with ICU mortality and their corresponding odds ratios for the entire cohort and subgroup, respectively; Tables S3 and S4 list the variables associated with ICU LOS and their corresponding odds ratios for the entire cohort and subgroup, respectively; Tables S5 and S6 list the variables associated with hospital mortality and their corresponding odds ratios for the entire cohort and subgroup, respectively; Tables S7 and S8 list the variables associated with hospital LOS and their corresponding odds ratios for the entire cohort and subgroup, respectively; Tables S9 and S10 list the variables associated with the likelihood of an invasive procedure being performed and their corresponding odds ratios for the entire cohort and subgroup, respectively; and Tables S11 and S12 list the variables associated with the likelihood of changing a patient's code status to DNR and their corresponding odds ratios for the entire cohort and subgroup, respectively. Click here for file
