# Supplementary material for: Intensivists' base specialty of training is associated with variations in mortality and practice patterns
Source: Crit Care. 2009 Dec 29;13(6):R209. doi: 10.1186/cc8227 (PMC2811951; doi:10.1186/cc8227)
Supplement: Additional file 1 — is available with the online version of this paper; it contains a total of 12 tables providing more detailed results of the analyses. Tables S1 and S2 list the variables associated with ICU mortality and their corresponding odds ratios for the entire cohort and subgroup, respectively; Tables S3 and S4 list the variables associated with ICU LOS and their corresponding odds ratios for the entire cohort and subgroup, respectively; Tables S5 and S6 list the variables associated with hospital mortality and their corresponding odds ratios for the entire cohort and subgroup, respectively; Tables S7 and S8 list the variables associated with hospital LOS and their corresponding odds ratios for the entire cohort and subgroup, respectively; Tables S9 and S10 list the variables associated with the likelihood of an invasive procedure being performed and their corresponding odds ratios for the entire cohort and subgroup, respectively; and Tables S11 and S12 list the variables associated with the likelihood of changing a patient's code status to DNR and their corresponding odds ratios for the entire cohort and subgroup, respectively. [file cc8227-S1.doc]

**Additional Files**

Table S1: Variables associated with ICU Mortality following multivariate analysis (entire cohort)

| **Variable** | **OR** | **95%CI** |
| --- | --- | --- |
| Code Status DNR at ICU Discharge | 24.2 | 20.1-29.1 |
| Invasive Procedures Performed | 1.29 | 1.22-1.36 |
| APACHE II | 1.10 | 1.08-1.11 |
| Age | 1.01 | 1.00-1.01 |
| ICU Occupancy at Time of Discharge | 0.99 | 0.98-0.99 |
| ICU LOS | 0.82 | 0.78-0.86 |
| Admission Diagnosis  - Neurologic  - Trauma  - Gastrointestinal  - Poisoning  - Other | 1.60  1.0  0.56  0.44  0.19 | 1.16-2.20  --  0.37-0.84  0.24-0.80  0.09-0.34 |
| Specialty  -AGSEM  -Internal Medicine  -Pulmonary | 1.0  0.80  0.69 | --  0.58-1.09  0.52-0.93 |

ICU: Intensive Care Unit; Code Status DNR: Patient had a Do-not resuscitate order at time of discharge from ICU or death in ICU; APACHE: Acute Physiology and Chronic Health Evaluation Score; LOS: Length of Stay; AGSEM: Intensivists with base specialty training in Anesthesia, General Surgery or Emergency Medicine

Table S2: Variables associated with ICU Mortality following multivariate analysis (subgroup)

| **Variable** | **OR** | **95%CI** |
| --- | --- | --- |
| Code Status DNR at ICU Discharge | 27.0 | 19.3-37.7 |
| Invasive Procedures Performed | 1.42 | 1.25-1.62 |
| APACHE II | 1.15 | 1.12-1.18 |
| ICU Occupancy at Time of Discharge | 0.98 | 0.97-1.00 |
| ICU LOS | 0.44 | 0.36-0.55 |
| Admission Diagnosis  - Trauma  - Pulmonary  - Cardiovascular  - Gastrointestinal  - Poisoning  - Other | 1.0  0.54  0.42  0.28  0.21  0.04 | --  0.35-0.91  0.23-0.79  0.14-0.59  0.09-0.47  0.02-0.13 |
| Specialty  -AGSEM  -Internal Medicine  -Pulmonary | 1.0  0.94  0.87 | --  0.54-1.64  0.52-1.46 |

ICU: Intensive Care Unit; Code Status DNR: Patient had a Do-not resuscitate order at time of discharge from ICU or death in ICU; APACHE: Acute Physiology and Chronic Health Evaluation Score; LOS: Length of Stay; AGSEM: Intensivists with base specialty training in Anesthesia, General Surgery or Emergency Medicine

Table S3: Variables associated with ICU LOS following multivariate analysis (entire cohort)

| **Variable** | **Coefficient** | **95%CI** |
| --- | --- | --- |
| Invasive Procedures Performed | 0.62 | 0.56 – 0.68 |
| Code Status DNR at ICU Discharge | 0.56 | 0.31 – 0.81 |
| Age | 0.03 | 0.02 – 0.03 |
| Weeks of Service per Year | 0.02 | 0.00 – 0.04 |
| APACHE II | -0.07 | -0.08 – -0.05 |
| TISS | -0.08 | -0.09 – -0.05 |
| Specialty  -AGSEM  -Internal Medicine  -Pulmonary | 0  -0.07  0.11 | --  -0.35 – 0.21  -0.20 – 0.42 |

ICU: Intensive Care Unit; LOS: Length of Stay; Code Status DNR: Patient had a Do-not resuscitate order at time of discharge from ICU or death in ICU; APACHE: Acute Physiology and Chronic Health Evaluation Score; TISS: Therapeutic Intervention Scoring System; AGSEM: Intensivists with base specialty training in Anesthesia, General Surgery or Emergency Medicine

Table S4: Variables associated with ICU LOS following multivariate analysis (subgroup)

| **Variable** | **Coefficient** | **95%CI** |
| --- | --- | --- |
| Code Status DNR at ICU Discharge | 0.39 | 0.27 – 0.51 |
| Age | 0.01 | 0.01 – 0.02 |
| TISS | -0.02 | -0.02 – -0.01 |
| APACHE II | -0.03 | -0.04 – -0.03 |
| Admission Diagnosis  - Trauma  - Other | 0  -0.98 | --  -1.50 – -0.46 |
| Specialty  -AGSEM  -Internal Medicine  -Pulmonary | 0  0.01  0.07 | --  -0.12 – 0.14  -0.07 – 0.22 |

ICU: Intensive Care Unit; LOS: Length of Stay; Code Status DNR: Patient had a Do-not resuscitate order at time of discharge from ICU or death in ICU; TISS: Therapeutic Intervention Scoring System; APACHE: Acute Physiology and Chronic Health Evaluation Score; AGSEM: Intensivists with base specialty training in Anesthesia, General Surgery or Emergency Medicine

Table S5: Variables associated with Hospital Mortality following multivariate analysis (entire cohort)

| **Variable** | **OR** | **95%CI** |
| --- | --- | --- |
| Code Status DNR at ICU Discharge | 17.3 | 14.6-20.4 |
| APACHE II | 1.09 | 1.08-1.10 |
| Invasive Procedures Performed | 1.08 | 1.04-1.12 |
| Age | 1.01 | 1.00-1.01 |
| ICU LOS | 0.82 | 0.78-0.86 |
| Admission Diagnosis  - Neurologic  - Gastrointestinal  - Trauma  - Poisoning  - Other | 1.79  1.40  1.0  0.45  0.42 | 1.36-2.34  1.02-1.94  --  0.28-0.72  0.21-0.86 |
| Specialty  -AGSEM  -Internal Medicine  -Pulmonary | 1.0  0.90  0.88 | --  0.69-1.17  0.68-1.13 |

ICU: Intensive Care Unit; Code Status DNR: Patient had a Do-not resuscitate order at time of discharge from ICU or death in ICU; APACHE: Acute Physiology and Chronic Health Evaluation Score; LOS: Length of Stay; AGSEM: Intensivists with base specialty training in Anesthesia, General Surgery or Emergency Medicine

Table S6: Variables associated with Hospital Mortality following multivariate analysis (subgroup)

| **Variable** | **OR** | **95%CI** |
| --- | --- | --- |
| Code Status DNR at ICU Discharge | 16.1 | 12.2-21.3 |
| Invasive Procedures Performed | 1.31 | 1.19-1.44 |
| APACHE II | 1.13 | 1.11-1.15 |
| Age | 1.01 | 1.00-1.02 |
| Admission Diagnosis  - Trauma  - Cardiovascular  - Pulmonary  - Poisoning  - Other | 1.0  0.64  0.57  0.22  0.14 | --  0.41-0.99  0.37-0.88  0.11-0.43  0.05-0.37 |
| Specialty  -AGSEM  -Internal Medicine  -Pulmonary | 1.0  1.22  0.96 | --  0.80-1.84  0.64-1.44 |

ICU: Intensive Care Unit; Code Status DNR: Patient had a Do-not resuscitate order at time of discharge from ICU or death in ICU; APACHE: Acute Physiology and Chronic Health Evaluation Score; AGSEM: Intensivists with base specialty training in Anesthesia, General Surgery or Emergency Medicine

Table S7: Variables associated with Hospital LOS following multivariate analysis (entire cohort)

| **Variable** | **Coefficient** | **95%CI** |
| --- | --- | --- |
| Invasive Procedures Performed | 1.50 | 0.89 – 2.10 |
| Code Status DNR at ICU Discharge | 0.86 | 0.45 – 1.27 |
| ICU Occupancy at Admission | 0.12 | 0.04 – 0.20 |
| Age | 0.17 | 0.11 – 0.24 |
| TISS | -0.22 | -0.32 – -0.11 |
| Specialty  -AGSEM  -Internal Medicine  -Pulmonary | 0  1.17  0.69 | --  -1.72 – 4.07  -2.43 – 3.82 |

LOS: Length of Stay; Code Status DNR: Patient had a Do-not resuscitate order at time of discharge from ICU or death in ICU; ICU: Intensive Care Unit; TISS: Therapeutic Intervention Scoring System; AGSEM: Intensivists with base specialty training in Anesthesia, General Surgery or Emergency Medicine

Table S8: Variables associated with Hospital LOS following multivariate analysis (subgroup)

| **Variable** | **Coefficient** | **95%CI** |
| --- | --- | --- |
| Invasive Procedures Performed | 1.42 | 0.12 – 2.73 |
| Code Status DNR at ICU Discharge | 0.63 | 0.33 – 0.94 |
| Age | 0.24 | 0.16 – 0.33 |
| TISS | -0.25 | -0.42 – -0.09 |
| Specialty  -AGSEM  -Internal Medicine  -Pulmonary | 0  -1.08  -2.18 | --  -5.17 – 3.01  -6.67 – 2.30 |

LOS: Length of Stay; Code Status DNR: Patient had a Do-not resuscitate order at time of discharge from ICU or death in ICU; ICU: Intensive Care Unit; Code Status; TISS: Therapeutic Intervention Scoring System; AGSEM: Intensivists with base specialty training in Anesthesia, General Surgery or Emergency Medicine

Table S9: Variables associated with likelihood of invasive procedures being performed following multivariate analysis (entire cohort)

| **Variable** | **OR** | **95%CI** |
| --- | --- | --- |
| ICU LOS | 1.02 | 1.01-1.03 |
| TISS | 1.02 | 1.02-1.03 |
| APACHE II | 1.00 | 1.00-1.01 |
| Weeks of Service per Year | 0.99 | 0.98-0.99 |
| Code Status DNR at ICU Discharge | 0.92 | 0.90-0.95 |
| Admission Diagnosis  - Trauma  - Neurologic  - Pulmonary  - Cardiovascular  - Gastrointestinal  - Other  - Poisoning | 1.0  0.90  0.89  0.89  0.85  0.76  0.66 | --  0.85-0.95  0.84-0.94  0.85-0.94  0.81-0.90  0.70-0.83  0.61-0.71 |
| Specialty  -AGSEM  -Internal Medicine  -Pulmonary | 1.0  1.04  0.96 | --  1.00-1.08  0.92-1.0 |

ICU: Intensive Care Unit; LOS: Length of Stay; TISS: Therapeutic Intervention Scoring System; APACHE: Acute Physiology and Chronic Health Evaluation Score; Code Status DNR: Patient had a Do-not resuscitate order at time of discharge from ICU or death in ICU; AGSEM: Intensivists with base specialty training in Anesthesia, General Surgery or Emergency Medicine

Table S10: Variables associated with likelihood of invasive procedures being performed following multivariate analysis (subgroup)

| **Variable** | **OR** | **95%CI** |
| --- | --- | --- |
| ICU LOS | 1.03 | 1.01-1.04 |
| TISS | 1.02 | 1.02-1.02 |
| APACHE II | 1.00 | 1.00-1.01 |
| ICU Occupancy at Admission | 1.00 | 1.00-1.01 |
| Code Status DNR at ICU Discharge | 0.94 | 0.91-0.98 |
| Admission Diagnosis  - Trauma  - Other  - Poisoning | 1.0  0.89  0.76 | --  0.80-0.99  0.70-0.82 |
| Specialty  -AGSEM  -Internal Medicine  -Pulmonary | 1.0  1.03  0.94 | --  0.98-1.07  0.90-0.99 |

ICU: Intensive Care Unit; LOS: Length of Stay; TISS: Therapeutic Intervention Scoring System; APACHE: Acute Physiology and Chronic Health Evaluation Score; Code Status DNR: Patient had a Do-not resuscitate order at time of discharge from ICU or death in ICU; AGSEM: Intensivists with base specialty training in Anesthesia, General Surgery or Emergency Medicine

Table S11: Variables associated with likelihood of changing a patient’s code status to DNR following multivariate analysis (entire cohort)

| **Variable** | **OR** | **95%CI** |
| --- | --- | --- |
| APACHE II | 1.07 | 1.06-1.08 |
| Invasive procedures performed | 1.07 | 1.02-1.11 |
| Age | 1.03 | 1.02-1.03 |
| TISS | 1.01 | 1.00-1.02 |
| ICU Occupancy at Discharge | 0.99 | 0.98-0.99 |
| Admission Diagnosis  - Neurologic  - Trauma  - Poisoning | 2.02  1.0  0.34 | 2.49-2.74  --  0.16-0.70 |
| Specialty  -AGSEM  -Internal Medicine  -Pulmonary | 1.0  1.13  1.01 | --  1.02-1.24  0.78-1.29 |

DNR: Do-Not-Resuscitate; APACHE: Acute Physiology and Chronic Health Evaluation Score; TISS: Therapeutic Intervention Scoring System; ICU: Intensive Care Unit; AGSEM: Intensivists with base specialty training in Anesthesia, General Surgery or Emergency Medicine

Table S12: Variables associated with likelihood of changing a patient’s code status to DNR following multivariate analysis (subgroup)

| **Variable** | **OR** | **95%CI** |
| --- | --- | --- |
| Invasive procedures performed | 1.18 | 1.04-1.34 |
| APACHE II | 1.07 | 1.04-1.09 |
| Age | 1.02 | 1.01-1.03 |
| TISS | 1.02 | 1.02-1.02 |
| ICU Occupancy at Discharge | 0.99 | 0.98-0.99 |
| Admission Diagnosis  - Trauma  - Gastrointestinal  - Pulmonary  - Cardiovascular  - Poisoning | 1.0  0.51  0.50  0.40  0.22 | --  0.27-0.94  0.29-0.89  0.19-0.83  0.10-0.49 |
| Specialty  -AGSEM  -Internal Medicine  -Pulmonary | 1.0  1.38  0.92 | --  1.09-1.66  0.60-1.26 |

DNR: Do-Not-Resuscitate; APACHE: Acute Physiology and Chronic Health Evaluation Score; TISS: Therapeutic Intervention Scoring System; ICU: Intensive Care Unit; AGSEM: Intensivists with base specialty training in Anesthesia, General Surgery or Emergency Medicine
